# P300 Speller Performance Predictor Based on RSVP Multi-feature

**DOI:** 10.3389/fnhum.2019.00261

**Published:** 2019-07-30

**Authors:** Kyungho Won, Moonyoung Kwon, Sehyeon Jang, Minkyu Ahn, Sung Chan Jun

**Affiliations:** ^1^School of Electrical Engineering and Computer Science, Gwangju Institute of Science and Technology, Gwangju, South Korea; ^2^School of Computer Science and Electrical Engineering, Handong Global University, Pohang, South Korea

**Keywords:** BCI, ERP, P300 speller, performance variation, prediction, RSVP

## Abstract

Brain-computer interface (BCI) systems were developed so that people can control computers or machines through their brain activity without moving their limbs. The P300 speller is one of the BCI applications used most commonly, as is very simple and reliable and can achieve satisfactory performance. However, like other BCIs, the P300 speller still has room for improvements in terms of its practical use, for example, selecting the best compromise between spelling accuracy and information transfer rate (ITR; speed) so that the P300 speller can maintain high accuracy while increasing spelling speed. Therefore, seeking correlates of, and predicting, the P300 speller’s performance is necessary to understand and improve the technique. In this work, we investigated the correlations between rapid serial visual presentation (RSVP) task features and the P300 speller’s performance. Fifty-five subjects participated in the RSVP and conventional matrix P300 speller tasks and RSVP behavioral and electroencephalography (EEG) features were compared in the P300’s speller performance. We found that several of the RSVP’s event-related potential (ERP) and behavioral features were correlated with the P300 speller’s offline binary classification accuracy. Using these features, we propose a simple multi-feature performance predictor (*r* = 0.53, *p* = 0.0001) that outperforms any single feature performance predictor, including that of the conventional RSVP T1% predictor (*r* = 0.28, *p* = 0.06). This result demonstrates that selective multi-features can predict BCI performance better than a single feature alone.

## Introduction

Brain-computer interface (BCI) systems help people operate computers and machines using neurophysiological signals recorded in the brain without moving physically. BCIs based on electroencephalography (EEG) are used most commonly because EEG is relatively less expensive and more durable and may capture direct brain activity with high temporal resolution. One of the EEG’s features used most frequently in BCIs is event-related potentials (ERPs), which represent the time-locked electrical potentials in the event (Patel and Azzam, 2005). In ERP, a positive deflection with a time delay of approximately 250 ms to 750 ms after the onset of a rare and unexpected stimulus is referred to as P300 (Patel and Azzam, 2005; Polich, 2007). Because of its stability (Karniski and Blair, 1989), convenience (Fazel-Rezai et al., 2012), and replicability (Walhovd and Fjell, 2002), P300-based BCIs have been used widely, such as in a speller application (Farwell and Donchin, 1988), smart home controls (Guger et al., 2009b), and brain painting (Münßinger et al., 2010). The P300 speller is a type of brain writer that types characters using a P300 component derived from characters that flash randomly in a letter matrix (Farwell and Donchin, 1988). It achieves reasonable accuracy even among patients with amyotrophic lateral sclerosis (ALS), so that patient studies have also been conducted actively (Guger et al., 2009a; Nijboer et al., 2010; Mak et al., 2012; Riccio et al., 2013; Geronimo et al., 2016; Wolpaw et al., 2018).

Two phases are commonly required to use BCI systems: calibration and test phases. In the calibration phase, a reasonable amount of data is collected, and classifier weights are trained. Then, during the test phase, users can operate the BCI systems using the classifier trained in the calibration phase. One of the hurdles in BCI is that the classification performance varies from subject to subject, and even from session to session. Another problem is that some people cannot achieve the performance necessary to operate BCI systems (Guger et al., 2009a; Blankertz et al., 2010; Ahn et al., 2013). For these individuals, the meaningless calibration phase may be frustrating when they realize that they cannot operate the BCI system after a long period of training wearing an EEG cap, and their poor BCI aptitude is considered a critical problem that requires resolution. Therefore, researchers have studied the prediction of BCI aptitude for each specific BCI type to suggest types suitable according to each user’s aptitude; further, they have proposed various solutions to the performance variation problem by understanding neurophysiological characteristics (Mainsah et al., 2017) and modulating paradigm schema better (Sellers et al., 2006; Kaufmann et al., 2011). The existing literature on predicting BCI users’ aptitude has been designed primarily to achieve a deeper understanding of the BCI control mechanism and what affects BCI performance using various characteristics, such as EEG features (Lee et al., 2011; Mak et al., 2012; Halder et al., 2013), psychological characteristics (Kleih et al., 2010; Nijboer et al., 2010; Hammer et al., 2018), and other biological characteristics (Kaufmann et al., 2012). Pre-screening subjects during training who are expected to have a low chance of success can be used to predict BCI aptitude as an intermediate solution before performance variation problems are resolved fully. Moreover, one can use the predictors identified as targets in user training to increase performance. For example, in motor imagery BCI, previous studies have found that resting-state frequency band powers are quite useful in predicting BCI performance (Blankertz et al., 2010; Ahn et al., 2013). Further, one study found that the upper alpha frequency band correlates with SSVEP BCI performance and may enhance BCI performance by regulating the upper alpha power through neurofeedback training (Wan et al., 2016). This study illustrated the complete process, from predicting performance to enhancing it by regulating the predictor.

Predicting the P300 speller’s performance has been investigated using various features, such as heart rate variability (Kaufmann et al., 2012), an auditory oddball task ERP (Halder et al., 2013), psychological factors assessed by a questionnaire (Kleih et al., 2010; Nijboer et al., 2010; Kleih and Kübler, 2013; Baykara et al., 2016; Hammer et al., 2018), cognitive capabilities assessed using a behavioral test (Nijboer et al., 2010; Riccio et al., 2013; Geronimo et al., 2016), and frequency band powers (Lee et al., 2011; Mak et al., 2012). However, such studies have considered only a single feature type in the prediction, for example, a neurophysiological or psychological feature, or cognitive ability. One study proposed a predictive model for P300 speller performance using multiple EEG features, such as Root Mean Square (RMS) amplitudes and frequency band powers (Mak et al., 2012), but it used task-related features and EEG features only. Another study used emotional stability, which consists of multiple psychological characteristics (Hammer et al., 2018), but used only psychological features. Yet another study investigated a rapid serial visual presentation (RSVP) task in which the participants are instructed to detect a target in a stream, and the authors reported a positive association between cognitive ability (referred to as RSVP T1%) and the P300 speller’s offline accuracy in people with ALS (Riccio et al., 2013). It is known that detection accuracy in an RSVP task can serve as an index of selective attention related to updating temporally variable information (Di Lollo et al., 2005; Kranczioch et al., 2007; Riccio et al., 2013) that also may be related to the P300 speller’s performance (Riccio et al., 2013). This study reminded us of the necessity to investigate neurophysiological signals in the RSVP task, as it can measure cognitive capability (RSVP T1%) and neurophysiological characteristics simultaneously so that multiple features can be used to predict P300 speller performance. RSVP is one of the visual oddball paradigms that elicits ERP, the features of which are quite likely to be correlated with P300 speller performance. Indeed, a previous study found that an auditory oddball ERP was significantly related to visual and auditory P300 speller performance (Halder et al., 2013). Therefore, we assumed that the RSVP EEG features may be correlated with the P300 speller’s performance and expected that both cognitive ability (RSVP T1%) and neurophysiological features (ERP properties) would be useful in predicting its performance more accurately. With respect to the relation between ERP features and the speller’s performance, most studies have focused typically on ERP amplitude and latency, such as P300 amplitude/latency and N200 amplitude/latency (Halder et al., 2013). In our previous study (Won et al., 2018), we investigated the relationship between the RSVP’s P300 amplitude and latency and the speller’s performance and found a positive correlation between amplitude and performance, while latency showed a nonsignificant negative relation. In this work, we conducted a detailed investigation with the goal to find additional correlates of P300 speller performance. We found that trial variations in P300 amplitude and latency are possible, which indicates that maintaining a stable P300 amplitude or latency may be another key to P300 speller performance.

We note that this work is an extended version of a conference paper (Won et al., 2018) that was reported at the IEEE SMC 2018 (Miyazaki, Japan, October 2018). In this work, we investigated the RSVP paradigm by analyzing its neurophysiological characteristics, as well as its cognitive ability. First, we investigated the spatial patterns of RSVP features related to P300 speller performance by observing sub-group scalp topographical patterns. Then, the RSVP features’ predictability was compared, and multi-feature performance predictors were proposed using multiple characteristics to enhance predictability.

## Materials and Methods

### Experimental Design and Data Recording

**Figure 1:**
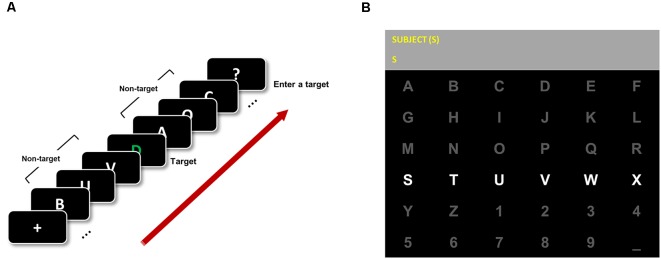
Rapid serial visual presentation (RSVP) and P300 speller application. **(A)** RSVP paradigm. All subjects were instructed to focus on a green character (target) in a white-character stream and enter the target character using the keyboard during a total of 40 trials. **(B)** The conventional 6 × 6 matrix speller was implemented in BCI2000 software.

A total of 55 healthy subjects participated in this experiment (41 males and 14 females, age: 23.93 ± 2.92 years). The experiment was conducted in a laboratory environment where the subjects sat in comfortable chairs approximately 1 m away from a 27″ monitor screen and faced straight ahead. EEG data were recorded at a 512 Hz sampling rate with a 32-channel system (BioSemi Active Two) using BCI2000 software during the RSVP and P300 speller tasks (Schalk et al., 2004), as shown in Figures 1A,B. The Institutional Review Board at Gwangju Institute of Science and Technology approved this experiment (20171106-HR-31-01-02), and all subjects were informed of all experimental procedures and signed written informed consents.

Two tasks were employed in this study—RSVP and P300 speller tasks. An RSVP task was conducted before the P300 speller task, and then the indices of temporal attention and physiological features were estimated and compared with the P300 speller’s performance (Kranczioch et al., 2007; Riccio et al., 2013). The detailed descriptions of each task are detailed below.

During the RSVP task, each target was embedded in a 21-character stream, and all stimuli were displayed in the center of the monitor screen on a black background at a 10 Hz refresh rate (Kranczioch et al., 2007; Riccio et al., 2013). A previous study (Riccio et al., 2013) recruited ALS patients with motor impairments and the operators were required to give a binary response (whether the target letter was a vowel or consonant) using their residual communication channels. However, in this study, we note that the subjects used the keyboard, rather than giving a binary response, as they all were healthy and residual communication channels were unnecessary. Before the task began, subjects were told that the target (T1) was green and the non-targets were white, and they were instructed to enter the target (T1) using the keyboard after each character stream was presented. In every trial, subjects were given 5 s to respond after each character stream passed. For example, a subject pressed “A”’ when s/he saw one green “A” in the character stream. In this study, the second target (T2) was not considered because a previous study found that T2 was not correlated with P300 speller performance or ERP (Riccio et al., 2013). The subjects performed a total of 40 experimental trials.

During the visual P300 speller task, subjects were asked to use a 6 × 6 matrix speller that contained letters (A-Z), digits (1–9), and spaces (_), as illustrated in Figure 1B. Each sequence consisted of 12 flashes (six columns and six rows) with a 125 ms flash duration followed by a 62.5 ms inter-stimulus interval (ISI). Fifteen repetitions (trials) per sequence were performed to enter a character. Thus, each target character flashed a total of 30 times (sum of column and row flashes), and non-target characters flashed a total of 150 times. This sufficient number of repetitions was chosen to ensure high accuracy and to observe changes in accuracy depending on the number of repetitions in future analyses. Each subject performed two calibration runs and four test runs. In the calibration runs, subjects were instructed to enter the words BRAIN and POWER without visual feedback because the classifier weights were not yet trained. P300 classifier weights were trained using BCI2000 software, which supports stepwise linear discriminant analysis (SWLDA), with calibration data (two runs) from each subject. To train the classifier weights, 800 ms from the stimulus onset was defined as an epoch and decimated to 20 Hz for 32 channels. Finally, the best 60 features among each feature vector were chosen. In the test runs, subjects were instructed to enter the pre-defined words SUBJECT, NEURONS, IMAGINE, and QUALITY. The characters selected were displayed in the second row of the speller application (see Figure 1B) during the test runs.

### RSVP Task T1% and ERP

**Figure 2:**
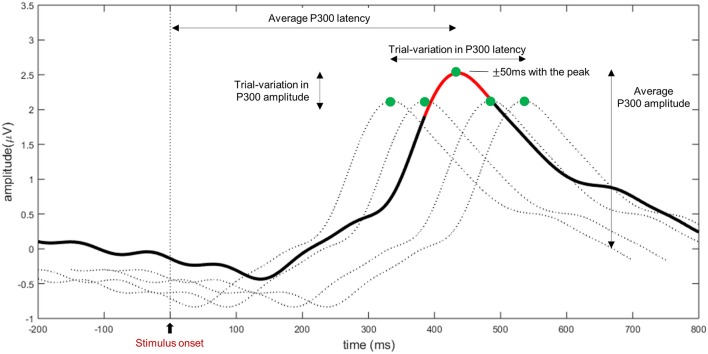
Event-related potential (ERP) features in the RSVP: P300 amplitude, P300 latency, trial variation in the P300 amplitude, and trial variation in the P300 latency. The bold line indicates the trial averaged P300, and dashed lines indicate single P300 trials (the same P300 response was presented for visual representation in this figure). For each electrode, P300 amplitude is defined as the time average of neighbor 50 ms with the peak amplitude (green dot: peak, red line: neighbor 50 ms with the peak), and the corresponding peak time point is regarded as the P300 latency. In addition, trial variations in the P300 amplitude and latency are defined as the inter-trial standard deviation of amplitude and latency, respectively.

RSVP T1% is defined as the accuracy in detecting the target (T1) in the RSVP task (cognitive ability), where a non-response is considered a wrong answer (Kranczioch et al., 2007; Riccio et al., 2013). To compute the ERP from the RSVP task, EEG data were first common average re-referenced and band-pass filtered through a 4th-order Butterworth filter with cut-off frequencies of 0.5 and 10 Hz. Then, artifacts such as eye blinks and eyeball movements were corrected with extended infomax independent component analysis (ICA) implemented in EEGLAB (Delorme and Makeig, 2004; Hoffmann and Falkenstein, 2008). Because the EEG data were recorded at 32 electrode channels, ICA decomposed the data into 32 component activations, which were categorized as EEG activity or artifacts by visual inspection of their scalp topographies, time series, and frequency spectra. The components categorized as artifacts were set to 0 when reconstructing the ICA-pruned data that were used in the analysis. Then, the cleaned data were divided into epochs of 800 ms from the stimulus onset, and a baseline correction was performed on the 200 ms preceding each epoch’s onset (Riccio et al., 2013). Among the epochs extracted, data with an amplitude greater than 100 μV or those exceeding the threshold (five times the standard deviation across trials) were rejected using the EEGLAB toolbox’s automatic removal features (Delorme et al., 2001; Delorme and Makeig, 2004). As the non-target and target epochs overlapped because of the fast refreshing rate, three non-target epochs adjacent to the targets were excluded from further analysis. After this pre-processing step, the following P300 components were calculated for the target conditions. First, P300 amplitude and latency for each epoch were calculated using the average of the channels selected (Fz, Cz, Pz, CP1, and CP2, which are associated with the fronto-parietal attention network nodes) to increase the signal-to-noise ratio (Gross et al., 2004; Siegel et al., 2008; Li et al., 2015). As Figure 2 shows, the P300 amplitude was defined as the average time of ±50 ms with the positive peak at each epoch, and the peak time point represented the P300 latency (Li et al., 2015). Moreover, we investigated the within-subject variation in P300 amplitude and latency (referred to hereinafter as trial variations in amplitude and latency) by observing the channel-averaged standard deviations of P300 amplitude and latency at the trial level.

### P300 Speller Offline Performance

In this study, the single-trial classification of distinguishing target vs. non-target was considered as P300 speller performance (Krusienski et al., 2006; Blankertz et al., 2011; Sharma, 2017). EEG data collected from the test runs were used to compute classification performance. The pre-processing step was the same as explained in section “RSVP Task T1% and ERP.” For each character, 30 target and 150 non-target epochs were extracted and yielded a total of 840 target and 4,200 non-target epochs, respectively. Then, the epochs extracted were down-sampled by averaging 24 time points without overlaps, which yielded a 32 × 17 feature vector for each epoch, where 32 was the number of electrode channels and 17 was the time points because there were 409 original time points for each channel (512 Hz × 0.8 s). Then, each feature vector was concatenated for classification. In this study, all electrode channels were used to train the classifier to investigate the feature space reduction from the entire head (covered with 32 channels) to specific areas.

SWLDA was selected to reduce the feature space from the concatenated feature vector by adding and removing features depending on their contribution to the classification labels (Krusienski et al., 2006; Sharma, 2017). The area under the ROC curve (AUC) was used for the performance metric as the classes were highly unbalanced (840 targets and 4,200 non-targets), which makes it unsuitable to use classification accuracy (Jeunet et al., 2018). The classification AUC was evaluated using 10-fold cross-validation. For each fold, the best 60 features from the training subsets were extracted to train classifier weights, and the AUC was calculated using the test subset. Finally, the average AUC of 10 folds was considered each subject’s P300 speller offline performance.

### Group Difference—RSVP ERP Features

We investigated the differences in ERP waveforms and scalp topography patterns in RSVP for high- and low-performance (P300 speller) groups and observed whether the features differed significantly. To identify clear between-group differences, we sorted the P300 speller performance in ascending order initially and considered the top 10 performers the high-performance group and the bottom 10 the low-performance group. The RSVP EEG spatial patterns (P300 amplitude and latency, trial variations in amplitude and latency) were group-averaged to investigate group differences, and their statistical significance was estimated using an unpaired Student’s *t*-test for the EEG features averaged over the electrodes selected (Fz, Cz, Pz, CP1, and CP2), as defined in section “RSVP Task T1% and ERP.”

### Association Between RSVP Task and P300 Speller and Proposed Multi-feature Predictor

From the observations in the high- and low-performance groups, we investigated the correlation between RSVP and P300 speller offline AUC. Because the RSVP task features and P300 speller AUC were distributed normally, we calculated Pearson’s correlation coefficients and their corresponding *p*-values to determine the coefficients’ statistical significance. First, the correlation between RSVP T1% and P300 speller performance was investigated as in a previous study (Riccio et al., 2013). In addition, P300 amplitude and latency and their trial variations in RSVP were used as the RSVP EEG features, and their correlations with P300 speller performance were investigated. The scalp topographies of the Pearson’s correlation coefficients between RSVP EEG features (P300 amplitude and latency and their trial variations) and P300 speller performance and the corresponding *p*-value topographies were plotted to emphasize which areas are likely to have high and low correlations.

To compare the predictability of P300 speller performance, we performed a linear regression analysis for each RSVP feature by setting a RSVP feature as the independent variable and P300 speller performance as the dependent variable. Each predictor (predictive P300 speller performance) was estimated using leave-one-subject-out cross-validation to avoid the overfitting problem. For example, the features from s01 were excluded when the regression coefficients for s01 were calculated. Linear regression was performed in MATLAB.

A previous study suggested a potential multi-feature-based performance factor by combining simple linear relations (positive and negative) between relative power levels (RPLs) and motor imagery BCI performance (Ahn et al., 2013). This study motivated us to combine multiple RSVP features to predict P300 speller performance more accurately. As a result, we attempted to evaluate our observations and expressed them in the form of a weighted sum, as follows:

(1) $$Multi\text{-}feature\;predictor=W_{1}X_{1}+W_{2}X_{2}+\cdots ,+W_{5}X_{5}$$

The observations’ weighted sum was regarded as a *Multi-feature predictor* that contains multiple RSVP features, including RSVP T1% (*X*_1_), P300 amplitude (*X*_2_), P300 latency (*X*_3_), trial variation in P300 amplitude (*X*_4_), and trial variation in P300 latency (*X*_5_). All of the RSVP features were z-scored before they were combined because they had different units. Two *Multi-feature predictor* models were proposed; simple multiple linear regression (regular model) and stepwise linear regression (stepwise model). Multiple linear regression and stepwise linear regression were performed using functions implemented in MATLAB. We evaluated the predictability of P300 speller performance for the *Multi-feature predictor* models given and compared it with predictors that used a single feature alone.

### Optimal RSVP Task Length

In this study, subjects performed the RSVP task during a total of 40 trials, which resulted in a total task duration of approximately 5 min, including response time. We investigated the way the correlations with P300 speller performance changed depending on the number of trials to identify the optimal task length. The Pearson’s correlation coefficients and their corresponding *p*-values between RSVP EEG features (P300 amplitude and latency, and their trial variations) were calculated, expanding the range of trials from the 5th to 40th from the first RSVP trial, such that each RSVP EEG feature and each correlation with P300 speller performance was calculated using the 1st to Nth RSVP trials. The minimum number of trials was set to five, as trial variations in P300 amplitude and latency were calculated using the standard deviation. Because bad trials were rejected in the pre-processing step, the remaining number of RSVP trials differed for every subject. Therefore, when calculating correlations using the 1st to Nth RSVP trials, if fewer than N trials remained, the 1st to the RSVP trials remaining were used to calculate correlations.

## Results

**Figure 3:**
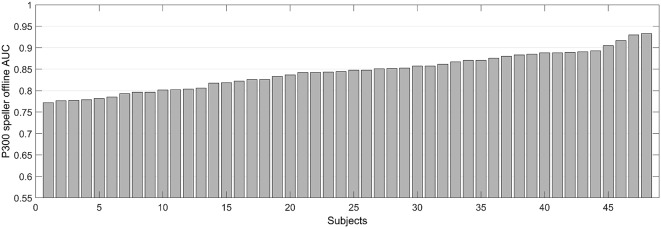
P300 speller offline area under the ROC curve (AUC). This indicates sorted P300 speller offline performance (single-trial-based binary classification) that was estimated by 10-fold cross-validation.

The following analyses were performed in this study with 48 subjects only, as five subjects’ keyboard responses were not recorded because the device malfunctioned during RSVP, and two subjects were identified as outliers, as their P300 speller offline performance (AUC: 0.6613 and 0.6924) was less than 2.5 standard deviations of their performance overall, which can indicate that they were less engaged in this study. Indeed, one subject failed to extract a P300 classifier weight with two calibration runs during the online experiment, so he performed several additional calibration runs to extract a classifier weight and finally achieved 39% online accuracy. The other subject indicated on the questionnaire that he thought he could not achieve an accuracy higher than 50% on every test run, but ultimately, he achieved 61% online accuracy. To clarify effects of these two outliers, the results were tested for both conditions—with/without outliers. In addition, 14.51% of the epochs were discarded as bad data. The grand-averaged waveform is presented in Figure 2, which shows the positive deflection in the target condition. The mean of P300 speller performance (offline AUC) was 0.84 ± 0.04 (Figure 3). In the RSVP task, the mean of RSVP T1% was 91.87% ± 5.7%, P300 amplitude was 2.88 μV ± 0.92 μV, and P300 latency was 455.8 ms ± 65.6 ms. The trial variations in P300 amplitude and latency were 1.88 μV ± 0.41 μV and 251.2 ms ± 26.6 ms, respectively. RSVP T1% showed a very narrow performance distribution, probably because subjects performed a small number of trials and the task was too simple for healthy subjects.

### Group Difference—RSVP ERP Features

**Figure 4:**
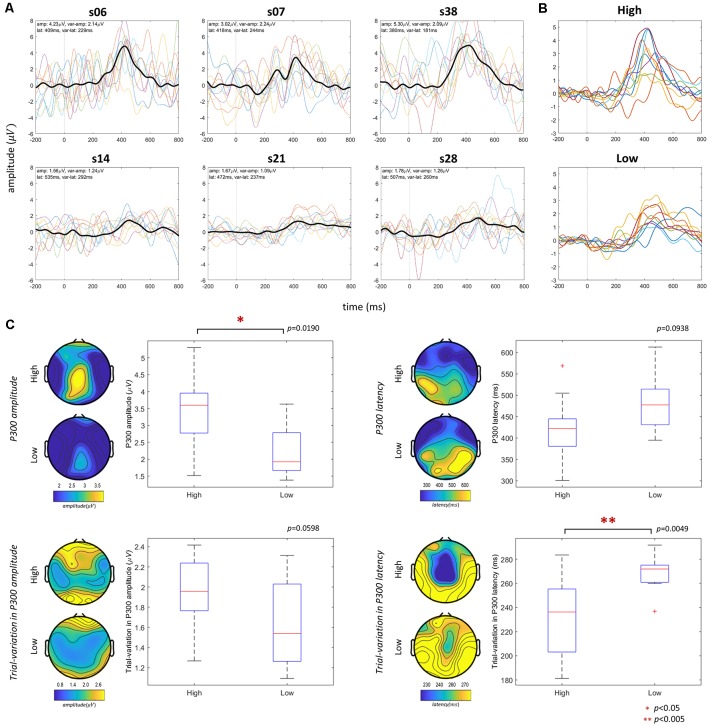
RSVP features of high and low performers. Panel **(A)** shows three representative waveforms (black bold line) from the top 10 and bottom 10 performers with their single-trial waveforms: thin dashed lines (for visual representation, 10 single-trial waveforms selected randomly were drawn). Panel **(B)** indicates ERP waveforms of all of the high and low performers. Panel **(C)** compares group-difference through the scalp topography maps representing the averaged spatial patterns of the RSVP electroencephalography (EEG) features for high and low performers, and the box plots show the statistical difference between high and low performers. The red asterisk denotes statistical significance as shown at the bottom. To compute spatial patterns (scalp topography) and group differences (box plot), single-electrode features and averaged electrode (Fz, Cz, Pz, CP1, and CP2) features were used, respectively.

**Figure 5:**
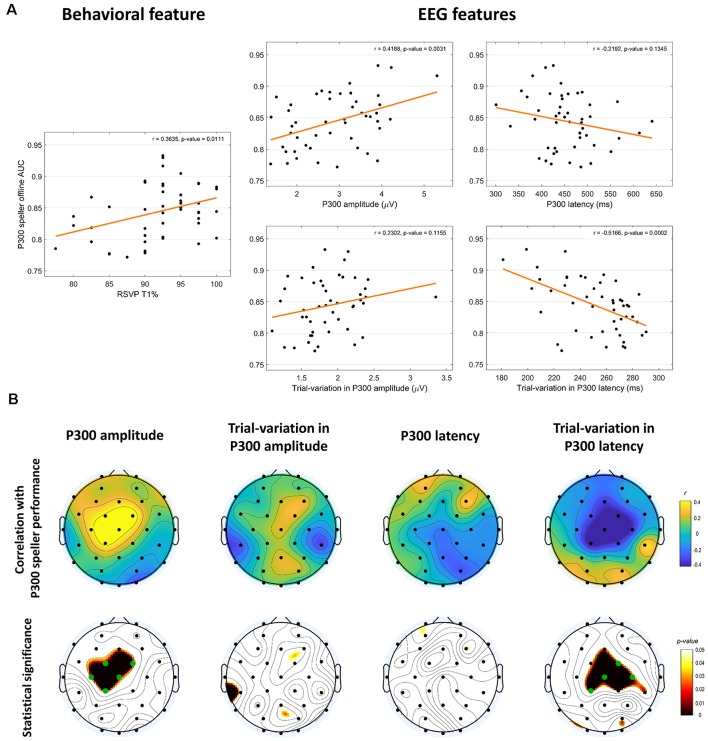
Association with P300 speller performance. **(A)** Relationship between the various RSVP features and P300 speller performance using Pearson’s correlation coefficients and linear regression analysis. RSVP T1% is a behavioral feature and the others are RSVP EEG features. All except P300 latency and trial variation in P300 amplitude were correlated significantly with P300 speller performance. **(B)** The spatial patterns of the relationship between RSVP EEG features and P300 speller performance. Pearson’s correlation coefficients and their statistical significance (uncorrected *p*-values) are presented in the top and bottom row, respectively. The green dots in the scalp topography in the bottom row denote significant channels after FDR-correction.

We investigated the difference between the RSVP ERP and EEG features in the P300 speller high- and low-performance groups (top 10 and bottom 10 performers, as defined in the Group difference—RSVP ERP features section), as illustrated in Figures 4A–C. We selected three subjects from both the high and low performance groups arbitrarily and illustrated their ERPs with single-trial waveforms to compare ERP shapes and properties, and found clear differences in their ERPs (Figures 4A,B): approximately 250 ms to 600 ms from the stimulus onset, the high performers (s06, s07, and s38) showed higher positive deflection, and their single-trial waveforms were time-locked at approximately 250 ms to 600 ms compared to those of the low performers (s14, s21, and s28). All of the high and low performers’ ERPs showed that high performers had higher and narrower positive deflections during RSVP compared to low performers (Figure 4B). Further, RSVP EEG features’ scalp topographical patterns were investigated in both groups. As Figure 4C shows, the high-performance group had a higher P300 amplitude in the central/parietal areas, which is consistent with the spatial patterns correlated with P300 speller performance described in Figure 5B; there also was a statistically significant between-group difference (*p* < 0.05). The spatial patterns in P300 latency did not differ significantly (*p* > 0.05), although there seemed to be a weak trend in which the high-performance group had a shorter latency than the low-performance group did. With respect to trial variations in P300 amplitude, the high-performance group had a slightly greater, but nonsignificant, variation in the midline (fronto-central and parieto-occipital) of the brain compared to the low-performance group (*p* > 0.05). A notable between-group difference was found in trial variation in P300 latency. In the scalp topography, the high-performance group showed lower trial variation in P300 latency in the central areas than did the low-performance group (*p* < 0.005: Figure 4C), indicating that the trial variation in P300 latency was associated negatively with P300 speller performance.

### Associations With P300 Speller Performance

We observed several relationships between RSVP features and P300 speller performance, as shown in Figure 5A. With respect to behavioral scores, a statistically significant positive correlation was found between RSVP T1% and P300 speller performance (*r* = 0.36, *p* < 0.05 without outliers, *r* = 0.30, *p* < 0.05 with outliers), indicating that the subjects with higher RSVP T1% achieved higher P300 speller performance, which is consistent with the results a previous study reported (Riccio et al., 2013). In the RSVP, a statistically significant positive correlation was found between the P300 amplitude and P300 speller performance (*r* = 0.42, *p* < 0.005 without outliers, *r* = 0.38, *p* < 0.01 with outliers), showing that the subjects with a higher P300 amplitude during the RSVP task achieved better performance, while the P300 latency in the RSVP and P300 speller performance showed a negative, but not statistically significant association (*r* = −0.22, *p* > 0.05 without outliers, *r* = −0.24, *p* > 0.05 with outliers). For trial variation in P300 components, we found a positive, but nonsignificant trend between trial variation in P300 amplitude and P300 speller performance (*r* = 0.23, *p* > 0.05 without outliers, *r* = 0.26, *p* > 0.05 with outliers), and an extremely negative correlation between trial variation in P300 latency and P300 speller performance (*r* = −0.52, *p* < 0.0005 without outliers, *r* = −0.52, *p* < 0.0005 with outliers).

Further, the scalp topographical distributions for the Pearson’s correlation coefficients between RSVP EEG features and P300 speller performance are illustrated in Figure 5B, and each correlation’s statistical significance is depicted in its corresponding *p*-value topography map (bottom row in Figure 5B, uncorrected); the FDR-correction was applied to present the statistical significance when multiple testing effects were minimized (Yoav and Yosef, 1995). For a better visual representation, the false discovery rate, q, was set to 0.1, and significant electrodes are displayed as green dots in the *p*-value topography maps in Figure 5B.

Overall, most statistically significant correlations disappeared after the FDR correction, implying that they were weak. Thus, the uncorrected *p*-value maps were used to investigate the spatial behaviors. First, in the relation between RSVP P300 amplitude and P300 speller performance, there were high positive correlations in the frontal/central areas that were highly statistically significant in the central area. With respect to P300 latency, the correlations overall were similar to those observed in section “Group Difference—RSVP ERP Features” (although nonsignificant). The trial variation in P300 amplitude showed positive correlations with P300 speller performance in the central/parieto-occipital areas, as observed in section “Group Difference—RSVP ERP Features” (although nonsignificant), although a single electrode showed an inverse relation. For the trial variation in P300 latency, there were strong negative correlations in the fronto-central areas that were highly statistically significant in the central area. As a result, the brain areas that showed statistically significant correlations with P300 speller performance were similar to those of the high- and low-performance group difference (Figure 4C).

To compare the predictability of the P300 speller with the RSVP features, we performed a regression analysis that was evaluated using leave-one-subject-out cross-validation, as explained in section “Association Between RSVP Task and P300 Speller and Proposed Multi-feature Predictor.” RSVP T1% yielded an *F*_df_ = 3.85 (*p* = 0.06 > 0.05, MSE = 16.55) without outliers, and an *F*_df_ = 2.27 (*p* = 0.14 > 0.05, MSE = 26.88) with outliers, the P300 amplitude of the RSVP yielded an *F*_df_ = 5.66 (*p* < 0.05, MSE = 15.96) without outliers, and an *F*_df_ = 3.84 (*p* = 0.06 > 0.05, MSE = 26.10) with outliers, while the RSVP P300 latency and trial variation in P300 amplitude were not statistically significant. Trial variations in latency yielded an *F*_df_ = 12.19 (*p* < 0.005, MSE = 14.11) without outliers, and an *F*_df_ = 12.66 (*p* < 0.001, MSE = 22.16) with outliers. The quantitative results, including the Pearson’s correlation coefficients (R), corresponding *p*-values, F-statistics, *R*^2^ values, and mean squared error (MSE) that were calculated as the difference between actual and estimated P300 speller performance, are tabulated in Table 1. When calculating MSE, the estimated and actual AUCs were multiplied by 100 because the AUC’s scale was too small (0–1) to calculate and compare MSE. According to these observations, we believe that the RSVP EEG features are able to predict P300 speller performance more accurately than its behavioral features are and we can combine the multiple features from the RSVP to predict P300 speller performance.

**Table 1:** The predictability of P300 speller for single- and multi-feature predictors.

	RSVP T1%	P300 amplitude	P300 latency	Trial-var. in P300 amplitude	Trial-var. in P300 latency	Multi-feature regular model	Multi-feature stepwise model
*R*	0.2781	0.3309	0.0714	0.0829	0.4578	**0.4775**	**0.5272**
*p*-value	0.0557	0.0216	0.6299	0.5753	0.0011	**0.0006**	**0.0001**
*F*	3.8547	5.6561	0.2354	0.3184	12.1943	**13.5826**	**17.7106**
*R*^2^	0.0773	0.1095	0.0051	0.0018	0.2095	**0.2280**	**0.2780**
*MSE*	16.5453	15.9595	18.1375	18.1510	14.1063	**14.0528**	**12.9125**

*The bold values indicate that they are quantified values from multi-feature predictors, whereas the plain values indicate that they were quantified values from single-feature predictors*.

### Multi-feature Performance Prediction

**Figure 6:**
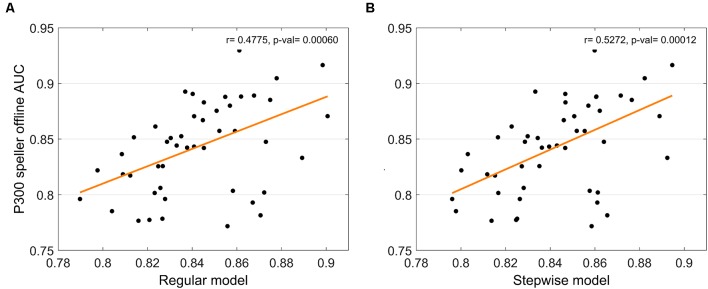
Relation between *multi-feature predictors* and P300 speller performance. The relationship with P300 speller performance are displayed for the *multi-feature regular model* **(A)** and *stepwise model* **(B)**, respectively.

We proposed *multi-feature predictors* with multiple linear regression and stepwise linear regression by combining the multiple features of the RSVP as in Equation (1). The *multi-feature regular model* yielded an *F*_df_ = 13.58 (*p* < 0.001, MSE = 14.05) without outliers, and an *F*_df_ = 11.97 (*p* < 0.005, MSE = 22.93) with outliers, while the *multi-feature stepwise model* yielded an *F*_df_ = 17.71 (*p* < 0.0005, MSE = 12.91) without outliers, and an *F*_df_ = 12.79 (*p* < 0.001, MSE = 22.35) with outliers. We observed that the *multi-feature regular model* (*r* = 0.48, *p* = 0.0001 without outliers, *r* = 0.45, *p =* 0.001 with outliers) and *multi-feature stepwise model* (*r* = 0.53, *p* = 0.0001 without outliers, *r* = 0.46, *p* = 0.0008 with outliers) performed better with respect to correlation strength and statistical significance compared to single feature predictors (see Table 1 and Figure 6), indicating that using multiple features is more effective than is using a single feature.

### Optimal RSVP Task Length

**Figure 7:**
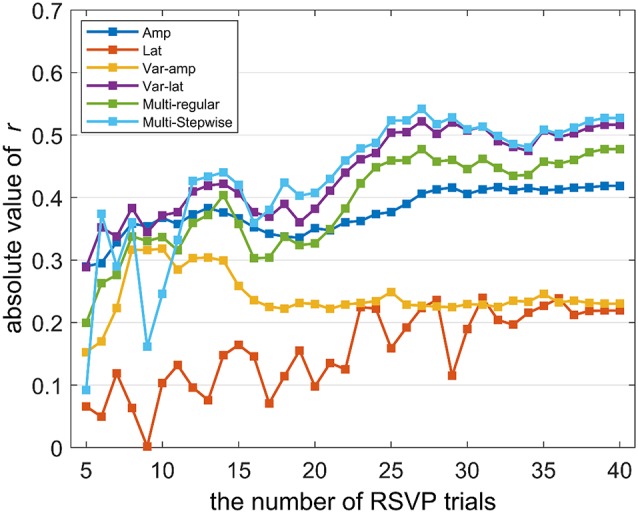
The correlation changes over the number of trials. This represents the way correlations change when the number of RSVP trials used to calculate EEG features varies. The X-axis represents the number of trials (1st to Nth RSVP trial), and the Y-axis represents the Pearson’s correlation coefficient’s absolute value.

The correlation changes between RSVP EEG features (P300 amplitude and latency, and their trial variation) and P300 speller performance were investigated as the number of RSVP trials varied (Figure 7). Further, the *multi-feature regular model* and *stepwise model* were investigated with the same method. P300 latency and its trial variation showed no statistically significant correlations with P300 speller performance. For the other features with statistically significant correlations with speller performance (P300 amplitude, trial-variation in P300 latency, and two multi-feature predictor models), we observed that the correlation strength was marginal after 25 RSVP trials. From this result, it can be expected that just approximately half of 50 RSVP trials may be sufficient to predict P300 speller performance.

## Discussion

The purpose of this study was to explore new correlates with P300 speller performance in an RSVP task in depth. A previous study found a statistically significant positive correlation between RSVP T1% and P300 speller offline accuracy in eight ALS patients (Riccio et al., 2013). T1’s detection rate in the RSVP can represent an index of selective attention, which indicates the ability to discriminate a target among distractors (Riccio et al., 2013). Motivated by this study, we investigated the relationship between the RSVP features and P300 speller performance further. In addition to RSVP T1%, which derives from behavior analysis, we explored neurophysiological characteristics with EEG data. The results showed that several of the RSVP task’s features were statistically significantly correlated with P300 speller performance, and similar relations were found in a between-group analysis. The RSVP P300 amplitude and latency showed positive and weak negative associations with P300 speller performance, respectively (Figure 5A). ERP amplitude and latency are known to be related to cognitive processing’s strength and timing, and a higher amplitude and shorter latency may lead to efficient cognitive processing (Duncan-Johnson and Donchin, 1982; Polich and Kok, 1995; Kok, 2001). Therefore, we believe that our observations are quite consistent with previous studies (Duncan-Johnson and Donchin, 1982; Polich and Kok, 1995; Kok, 2001) and that the relationship shown in Figure 5A are related to efficient cognitive processing.

With respect to inter-trial variations in P300 amplitude and latency, we hypothesized that maintaining stable cognitive processing during a cognitive task might affect P300 speller performance strongly. Hence, we quantified the maintenance level simply by estimating the standard deviation of the P300 amplitude and latency over trials and investigated their relationship with P300 speller performance to confirm our hypothesis. The trial variation in P300 latency was correlated negatively with P300 speller performance, indicating that subjects with a more stable P300 latency achieved better performance. However, in contrast to our hypothesis, the trial variation in P300 amplitude was related positively with P300 speller performance, although it was not significant; thus, we inspected the relation between P300 components and their trial variations to clarify whether high P300 components may cause high trial variation. We found that the trial variations in P300 amplitude and latency were correlated positively and significantly with the corresponding P300 components (*r* = 0.61, *p* < 0.00001 for amplitude and *r* = 0.29, *p* < 0.05 for latency), indicating that the high P300 components are likely to cause high trial variation and low P300 components may cause low trial variation. However, these results may be attributable to small abnormalities, such as outliers. Because a small number of trials was used in the analysis, precise interpretation may be made only with a sufficiently large number of trials, which will be addressed in future work. Another possible explanation for the positive associations between trial variation in P300 amplitude and P300 speller performance may derive from discrimination against non-target responses, in that P300 is derived from the responses to target events that show greater brain activity than responses to non-target events (Patel and Azzam, 2005; Polich, 2007), and thus, it may lead to higher trial variation in P300 amplitude. In addition, it is noteworthy that trial variation in P300 latency demonstrated a higher correlation with P300 speller performance (*r* = −0.52) than did P300 latency (*r* = −0.22). We inferred from this result that a critical correlate of P300 speller performance actually is the ability to stabilize cognitive processing rather than rapid cognitive processing itself. Hence, new feedback training protocols that focus on locking reactive timing against stimuli may be proposed to enhance ERP-based BCI.

**Figure 8:**
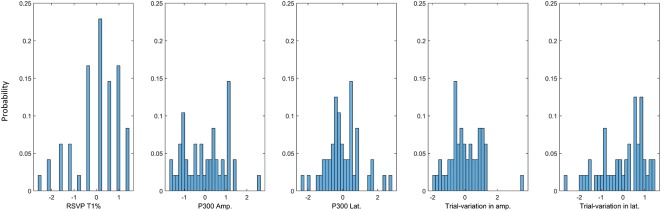
Data distribution of each RSVP feature. The distribution of each of the RSVP features is depicted using relative frequency histograms (*N* = 48). Compared to the other RSVP features, RSVP T1% showed a sharp and sparse distribution.

We used several RSVP features to investigate their associations with P300 speller performance. As Figure 5A shows, the RSVP P300 amplitude and trial variation in P300 latency (neurophysiological features) predicted P300 speller performance more accurately than did RSVP T1% (behavioral feature). In addition, when the distribution of each *z*-scored feature was expressed using a relative frequency histogram (Figure 8), RSVP T1% showed a very narrow and sharp distribution compared to the neurophysiological features, and this distribution is expected to yield biased results, depending on the various performance measures. We inferred that the RSVP task may be too simple for healthy subjects, and the RSVP T1% may become saturated easily, particularly when there is an insufficient number of trials. On the other hand, the neurophysiological features showed dense distributions under the same condition. Therefore, the neurophysiological features may be more robust than the behavioral feature (RSVP T1%) when a relatively small dataset is used. As proposed in the previous sections, the use of multiple features can be another solution. We observed already that by combining all of the RSVP features according to their relationship with P300 speller performance, a *multi-feature predictor model* could achieve higher predictability than any single feature predictor.

When predicting BCI performance, it may be skeptical to use tests in addition to the BCI system users intend to use. However, predicting the aptitude for the BCI system may frustrate users because they receive direct feedback whether or not they can use it. Instead, performance prediction using an indirect method can give flexibility and reduce users’ frustration by recommending other systems when user’s aptitude for the current BCI system is unsuitable. The reason to use the RSVP paradigm to predict the P300 speller’s performance is that RSVP is similar to the P300 speller, but it does not require gaze control, and the cognitive workload is much smaller (concentrating on a green-colored target letter in a 2s sequential letter stream), compared to the workload in the P300 speller (concentrating on a target character, counting how many times it flashes, and avoiding distraction from adjacent flashing rows/columns until the target character is selected). Further, the RSVP paradigm can illustrate both cognitive ability and neurophysiological characteristics simultaneously. Thus, it is believed that this additional information will improve the ability to predict P300 speller performance. Moreover, people can choose to use either their cognitive abilities or neurophysiological characteristics depending on the situation. For example, when potential users have residual communication channels other than brain signals, they can enhance predictability by combining cognitive ability and neurophysiological features. On the other hand, when residual communication channels are unavailable, they can use only neurophysiological features, or only cognitive ability if they want the prediction procedure to be simpler. Therefore, the RSVP task might be a suitable way to predict P300 speller performance. The neurophysiological characteristics common both to RSVP and the P300 speller can be demonstrated by comparing them with respect to speller applications. The RSVP paradigm often is regarded as a gaze independent speller (Treder et al., 2011; Chennu et al., 2013). These two spellers use the same P300 features. With respect to cognitive ability, several studies have found that detection accuracy of RSVP T1 may use to measure the level of selective attention (Kranczioch et al., 2007; Riccio et al., 2013). A previous study (Riccio et al., 2013) described selective attention as attentional filtering capacity, which refers to the basic ability to filter the object of interest from distractors temporally and maintain the filtering for a certain period of time. The concept of attentional filtering seems to match the P300 speller scheme. During P300 speller sessions, subjects must filter the target rows/columns from non-target rows/columns until a letter is entered, so attentional filtering capacity could explain this process. Therefore, the RSVP paradigm and P300 speller may share common characteristics in EEG and cognitive function that may be quite suitable to develop a multiple-feature predictor of P300 speller performance. Although it is unsurprising that RSVP EEG features are related to P300 speller performance because both involve the P300, this intuition may be erroneous sometimes and has not been demonstrated formally before. Similarly, Halder et al. (2013) investigated the relation between P300 elicited with the auditory oddball task and P300 speller performance. Although the auditory oddball task and P300 speller both involve the P300, confirming and quantifying how strongly P300 elicited with the auditory oddball correlated with P300 speller performance was meaningful. The results from this study confirmed empirically that the intuition is true using a sufficient number of subjects and quantifying the relationship between various features, which is new, and could be useful both in advancing neuroscientific knowledge and informing future BCI studies.

According to the RSVP features’ scalp topographical distributions and their correlations, most features associated with P300 speller performance were observed in the centro-parietal areas. This result is consistent with the literature on the centro-parietal P3b component, which is known to reflect memory storage and serves to link stimulus characteristics and attention (Patel and Azzam, 2005; Riccio et al., 2013). Because we focused on ERP properties in this work, these spatial patterns (Figures 4, 5) show the most interesting areas in which clear and robust P300 waveforms may be obtained during the RSVP. These spatial patterns also are used to choose the electrode channels for channel averaging or Principle Component Analysis (PCA) when the RSVP paradigm is used.

With respect to RSVP task length, we observed that using approximately half of the total trials (approximately 25) showed similar strong correlations and can reduce the time required for prediction by approximately half of the current RSVP settings. However, we should investigate this result with a greater number of trials to choose the number of RSVP trials that predicts P300 speller performance best. Moreover, the RSVP task length could be reduced greatly by reducing response time. In this study, as a fixed response time (5s) was given to all subjects regardless of their button responses, the response time could be adjusted depending upon each subject’s button response speed to reduce RSVP task length.

In this study, we investigated the correlations between the RSVP and P300 speller performance further by identifying RSVP’s EEG features, comparing a behavioral feature and neurophysiological features’ predictability, and combining such RSVP features. As a result, we found that some neurophysiological features predicted P300 speller performance better and had a dense distribution compared to the behavioral feature, even with a small number of trials. Because all analyses in this work were performed using a relatively small number of trials, more detailed investigation should be made by varying the number of trials (from sufficiently large to quite small) for sounder and more reliable interpretation. Further, the standard deviations of P300 amplitude and latency at the trial level need to be inspected extensively with a sufficiently large number of trials to determine the way in which they depend on the P300 component’s magnitude. The multi-feature performance predictor (*multi-feature regular and stepwise model* proposed here) predicted P300 speller performance more accurately than did any single feature predictor. We expect that prediction may be improved further by selecting optimal electrode channels. In this study, only five electrodes (Fz, Cz, Pz, CP1, and CP2) were used for channel-averaged ERP waveforms to increase the SNR. Because averaging multiple electrode channels likely decreases SNR, electrode channels should be selected more carefully. Alternatively, a single electrode channel (Riccio et al., 2013; Hammer et al., 2018) or PCA (Lee et al., 2011; Elsawy et al., 2014; Sharma, 2017) may be chosen. In addition, to enhance predictability, the optimization methods used to combine multiple features should be explored using analyses other than linear regression.

We believe that trial-by-trial couplings are quite interesting and may be considered additional RSVP features. For example, inter-trial coherence (ITC) can capture trial-by-trial coupling better than can trial variation in P300 components because it does not depend on the magnitude, but on phase locking at the trial level instead (Debener et al., 2005). In addition, a more detailed investigation of the performance predictor can be conducted by changing the electrode channels and using various performance measures, such as letter detection accuracy and information transfer rate (ITR).

One limitation of this study was the use of offline results. We used sufficiently long parameter settings in this study to ensure that every subject could achieve reasonable accuracies, such as 15 repetitions for each letter, a relatively longer flash duration (125 ms) and ISI (62.5 ms), and a long calibration phase (2 runs). Under these experimental conditions, more than 40 subjects achieved >90% accuracy, which led to less meaningful results [a ceiling effect of online visual P300 BCI performance reported in a previous study (Halder et al., 2013)]. As an alternative, we used single-trial classification AUC, which is more similar to P300, as the P300 speller is indeed the summation of single-trial classification results (Blankertz et al., 2011). To address this limitation, various performance measures, such as the number of repetitions required to detect a particular letter accurately, which is related to spelling speed and offline letter detection accuracy depending on the number of trials, can be considered alternatives to online accuracy. Further, a new experiment can be performed with moderate parameter settings by reducing the calibration phase and speeding up the P300 speller, which we will investigate in the future.

Another limitation is that the subject must wear an EEG cap to predict P300 speller performance; thus, EEG-based P300 speller performance predictors may not be very practical, as wearing an EEG cap is inconvenient and time-consuming. However, as shown in Figures 6, 8, using electrophysiological and behavioral features combined enhanced stability and predictability dramatically. Therefore, such enhanced stability and predictability may compensate for the inconvenience of wearing an EEG cap. To minimize the inconvenience, a considerable reduction in the number of electrodes (perhaps a single electrode) or use of dry electrodes may be considered. In this study, five electrodes (Fz, Cz, Pz, CP1, and CP2) were used to calculate the performance predictors, but a single electrode may be considered for scalp topographic patterns, as they yielded trends nearly the same (not shown here) as those in Figures 4C, 5B. Comparing convenience and predictability using several cognitive tasks, such as an auditory oddball task (Halder et al., 2013), under the same conditions, also can be helpful in choosing the best cognitive task to predict P300 speller performance.

Overall, as with other prediction studies (Kaufmann et al., 2012; Halder et al., 2013; Riccio et al., 2013; Geronimo et al., 2016), this study helped clarify the cognitive and neurophysiological substrate associated with BCI control. Using a number of performance prediction studies, including this one, researchers or end users can test and recommend which BCI system is the best for a potential user group, or can attempt to enhance BCI performance by modulating specific substrates related to BCI performance using neurofeedback training (Wan et al., 2016) and brain stimulation approaches.

## Conclusion

We investigated the relationship between RSVP task features and P300 speller (offline) performance to explore existing and to compare new correlates of P300 speller performance in an RSVP task. We observed statistically significant correlations with P300 speller performance in a behavioral feature (RSVP T1%) and a neurophysiological feature (P300 amplitude). Moreover, a new correlate was found, P300 latency’s trial variation, which suggests that maintaining stable P300 may be an important key in P300 speller performance. A comparative analysis revealed that neurophysiological features predict P300 speller performance better than a behavioral feature, and multi-feature predictors generated by the correlations observed outperformed any single feature predictor.

## Ethics Statement

This study was carried out in accordance with the recommendations of the Institutional Review Board at Gwangju Institute of Science and Technology with written informed consent from all participants. All the participants gave the written informed consent in accordance with the Declaration of Helsinki. The protocol was approved by the Institutional Review Board at Gwangju Institute of Science and Technology (20171106-HR-31-01-02).

## Author Contributions

KW, MK, SJ, MA and SCJ designed the study. KW and SCJ collected data and contributed to the editing of the final manuscript. KW analyzed data and wrote the manuscript.

## Conflict of Interest Statement

The authors declare that the research was conducted in the absence of any commercial or financial relationships that could be construed as a potential conflict of interest.
